# Causal clarity in statistical software

**DOI:** 10.1093/ije/dyaf136

**Published:** 2025-07-21

**Authors:** Maurice N Korf, Nan Van Geloven, Jesse H Krijthe, Jeremy A Labrecque

**Affiliations:** Department of Epidemiology, Erasmus MC University Medical Center, Rotterdam, The Netherlands; Department of Biomedical Data Sciences, Leiden University Medical Center, Leiden, The Netherlands; Department of Intelligent Systems, Delft University of Technology, Delft, The Netherlands; Department of Epidemiology, Erasmus MC University Medical Center, Rotterdam, The Netherlands

## Introduction

Imagine running a simple regression in any statistical software of choice—but this time, you only get a point estimate of the regression coefficient. There is no standard error, no confidence interval, no *P*-value, and no diagnostics like the *F*-statistic or *R*^2^. Such limited software output would not be very useful: a point estimate alone, without any accompanying statistical inference, is inadequate for meaningful conclusions. Consequently, most statistical software currently provides, by default, additional information beyond the point estimate to support users in their interpretation. While certain considerations may still be absent from current statistical software output (e.g. modeling assumptions), it is at least worth recognizing that significant efforts have already been made over the past decades.

In contrast, when the goal is causal inference, software output to support this goal has been largely absent. For instance, when reviewing widely used statistical software for causal inference, we found that none explicitly report the underlying causal assumptions, and indicative diagnostics to facilitate the assessment of these assumptions are rarely provided either. Yet, providing a causal effect estimate without reference to the causal assumptions is incomplete reporting of a result, similar to how reporting just a point estimate for statistical inference does not provide enough information to judge a claim. This is not merely an observation confined to software reporting; in fact, it is intrinsically linked to the widespread underreporting of causal analyses in empirical research. Many examples have been described in which explicit causal methods were employed, yet the corresponding causal assumptions were ignored or insufficiently discussed [1–3]. Even when assumptions were mentioned, diagnostics were frequently absent or inappropriately evaluated. For example, studies applying inverse probability treatment weighting often do not inspect covariate balance or do so inappropriately [4, 5]. While there may undoubtedly be various explanations for this, the lack of support in software output is one likely contributing factor, which could be addressed effectively.

Providing information relevant for interpreting a causal effect estimate directly in the reported output has the potential to improve the quality and transparency of applied causal inference. Our goal is therefore to initiate a dialogue about desirable conventions concerning the output obtained from software for causal inference, particularly regarding what information should be reported to provide users the greatest support. In our opinion, relevant causal information that software should additionally report on includes: (i) the causal estimand, (ii) results from estimators that rely on different modeling assumptions, and (iii) the corresponding causal assumptions alongside relevant diagnostics. We will discuss each component in detail, explaining why we believe that reporting on it contributes to safer causal inference. To guide this discussion, we have developed an R function that demonstrates one possible implementation of this. We have called this function CarefullyCausal and it is available at: https://github.com/mauricekorf/CarefullyCausal. Before presenting our suggestions, we will first review typical output generated by software for causal inference to outline current practices.

## Review of software output in causal inference

Our review of popular causal inference functions in R, SPSS, STATA, and SAS revealed that the target estimand is generally not reported or only broadly defined, only a single estimator is typically considered, none explicitly mention the causal assumptions in the output, and relevant diagnostics are rarely provided by default (Supplementary Table S1, Supplementary section A–C). For example, in R, when using the tmle package [6] for targeted maximum likelihood estimation, which explicitly estimates causal effects, it returns almost exclusively information relevant to statistical inference. The only information relevant to causal inference is a label of the estimate and the range of the estimated propensity score. Although it labels the estimated effect as an additive treatment effect among the entire population (ATE), among the treated (ATT), or among the controls (ATC), it makes no mention of the conditions under which we can interpret the estimated quantities as such, nor are any relevant diagnostics reported. As another example, the mediation package [7] used for causal mediation analyses reinforces the general observation that no information relevant to causal inference other than the effect estimates is provided. However, on a positive note, it does offer an option to do sensitivity analyses for the existence of unobserved pre-treatment covariates, although no active call or mention of this is provided in the default output. Another encouraging aspect was identified in the ivreg package [8], used for instrumental variable regression, as it reports diagnostic tests by default (e.g. “weak instrument,” “Wu-Hausman,” and “Sargan”) along with their corresponding values. It remains, nevertheless, an exception rather than the rule, and apart from reporting those diagnostics, no further causal inference-related information was provided.

These observations are not necessarily specific to R software, but are also, to the best of our knowledge, true of SPSS, STATA, and SAS. For instance, base SPSS does not offer an explicit interface for causal analyses, and thus a user is required to follow interfaces designed for statistical inference only [9]. Since SPSS provides no support for explicit causal analyses, external macros have been developed for specific applications, such as the causal mediation macro by Valeri and VanderWeele [10]. However, despite these additions, the capabilities remain constrained by SPSS’s design, with causal analysis output limited to effect estimates. In contrast to SPSS, base STATA does offer a point-and-click interface for causal analyses. While this interface improves user intuitiveness for conducting causal analyses, it still omits the underlying causal assumptions both in the pre-analysis interface and the reported output. Moreover, although users can select in the interface whether to estimate the ATE, ATT, or potential-outcome means, no estimand is specified in the output, nor are diagnostics provided [11]. Similar to STATA, SAS provides functions specifically designed for causal analyses, encompassing not only treatment effect estimation but also the construction of causal graphs with features such as identifying valid adjustment sets. However, despite the range of available causal functionalities, the software’s reported output remains primarily oriented toward statistical inference. While some functions label estimates as ATE or ATT and provide access to diagnostic tools, the output does not systematically report the underlying causal assumptions, the specification of the estimand, estimates obtained from multiple estimators, or the contextual information necessary for interpreting the diagnostics [12].

Although we have primarily highlighted the general lack of support in software when estimating causal effects, we also wish to emphasize that there are exceptions, such as the *DoWhy* library [13], although it is designed for Python. In fact, we do not assert that there are no exemplary cases; rather, our point is that such examples are exceptions, not the rule, while we believe that this is something that should be encouraged. We pose there is an opportunity to advance the quality of applied causal inference through software by making it easier to do the right thing and making it harder to make mistakes [14].

## Software output of a causal analysis

Determining which information to include in the reported output requires balancing the need for sufficient detail with maintaining clarity and conciseness, while also accounting for software limitations. Considering this, we believe the following information to be both important and feasible to integrate: (i) the causal estimand, (ii) results from different estimators that rely on different modeling assumptions, and (iii) the causal assumptions alongside diagnostics. To demonstrate one possible way how this might be integrated in software, we present an example function, CarefullyCausal, and apply it to the NHEFS data [15, 16], where we hypothetically evaluate the effect of quitting smoking (qsmk) on weight change between 1982 and 1971 (wt82_71) while adjusting for a specified set of variables (see Supplementary section D for details). We start by executing the following minimal call in R:

Output <- CarefullyCausal(wt82_71 ∼ qsmk + race + sex + education + smokeintensity + smokeyrs + wt71 + exercise + active + age, data = df, exposure = "qsmk", family = "gaussian")

### The causal estimand

An estimand is a precise description of the target quantity that would answer the research question and should be decided on before the analysis, as it guides the study design, estimator selection, and informs whether the research question has been adequately answered. There are many available resources to help define an estimand (e.g. [17–20]), yet, even in the reporting of randomized trials, estimands remain for the most part poorly defined [21, 22].

Statistical software is designed to implement estimators and typically does not mention an estimand. This could be because, having chosen an estimator, it is assumed that the user is aware of their estimand. This could also be because many aspects of an estimand, particularly causal ones, cannot be inferred from the information provided to an estimator function. For example, the target population, duration and timing of exposure, the precise definition of the exposure, and the follow-up period [21] are among many aspects of the estimand that cannot be implied from the inputs to the estimator. However, some information about an estimand can still be inferred. If a user chooses a function that explicitly estimates a causal effect, this choice implies that the user is targeting a causal estimand. The user must directly input and therefore make explicit the outcome, exposure variable, and adjustment set. The choice of estimator will also imply an effect measure. Even if this information should already be known to the user, reporting even these aspects of an estimand in the software’s output can serve as a useful reminder of the estimand being targeted, particularly the fact that the estimand contains counterfactual quantities. Stating the causal estimand clearly may alleviate some confusion between predictive and causal questions observed in the literature [23]. This reminder may also help alleviate some of the taboo around using causal language in research questions [24], and it might prevent the use of associational language for the research question when the goal is clearly causal, such as is the case with the use of TMLE or mediation analyses [25, 26].

In short, we argue that reporting the causal estimand in software output can be of added value. In CarefullyCausal, this is reported on the first line of output and is inferred from a user’s input arguments, including the outcome of interest, the exposure variable, causal contrast, and adjustment set, as illustrated below.

Estimand: Average Treatment Effect

E[wt82_71^qsmk=1] - E[wt82_71^qsmk=0]

Adjustment set: race, sex, education, smokeintensity, smokeyrs, wt71, exercise, active, age

*Please see output at $Estimand_interpretation for details

### Results under different modeling assumptions

An estimator is the process used to get an estimate of the estimand from data. Different estimators can be used for similar causal estimands (e.g. standardization, g-estimation, inverse probability of treatment weighting), each relying on different modeling assumptions. In most statistical software functions, only a single estimator is implemented.

There are benefits to using more than one estimator to answer the same causal question. When the estimates from different estimators targeting the same estimand differ by an important amount, it is an indication that the assumptions for at least one estimator are violated. When such differences are observed between estimators, the user should investigate potential reasons for these disagreements. Of course, lack of substantial differences is no guarantee that the estimates are unbiased or that all models are well-specified, although it may sometimes be argued that the bias resulting from model misspecification is unlikely to be of the same magnitude and direction for all models [15]. By providing estimates that rely on different modeling assumptions, a more informed judgment can be formed as compared to when using a single estimator.

Therefore, we recommend that causal software, when possible, include estimates from multiple estimators that rely on different modeling assumptions. As an example, in CarefullyCausal, we have integrated the following estimators: outcome regression, inverse probability of treatment weighting, two different standardization approaches, and targeted maximum likelihood estimation. Given the different estimators, CarefullyCausal outputs a single table with the estimate of the average treatment effect from each estimator as well as the relevant information for statistical inference, such as the standard error, confidence interval, as well as *S*-values [27, 28] and *P*-values. The table of estimates does not include coefficients for the adjustment variables, which may help users avoid the Table 2 fallacy [29].

Treatment effect:

**Table:** 

	Estimate	Std. error	*P*-value	*S*-value	95% CI, lower	95% CI, upper
qsmk1 outcome regression	3.381	0.441	0.000	44.858	2.517	4.246
qsmk1 IPTW	3.318	0.494	0.000	35.198	2.351	4.286
qsmk1 S-standardization	3.381	0.426	0.000	Inf	2.599	4.268
qsmk1 T-standardization	3.448	0.474	0.000	Inf	2.514	4.371
qsmk1 TMLE	3.370	0.494	0.000	Inf	2.401	4.339

Reference exposure level: 0

Please evaluate whether the difference between the lowest estimate: 3.3183 and highest: 3.4482 is of substance, given the nature of the data. If so, evaluate the different modeling assumptions underlying each estimator.

### Causal assumptions and diagnostics

Causal studies inevitably target unobservable quantities (e.g. counterfactuals), and therefore we must always make untestable causal assumptions to draw causal conclusions from data. When the causal assumptions are not explicitly addressed in a study, a reader may be tempted to draw much stronger conclusions than is warranted. Some causal assumptions are almost always reported, e.g. no uncontrolled confounding, while others are less often mentioned, e.g. positivity and consistency [1, 30]. Confronting the user with these assumptions helps minimize the possibility that the user reports their results without at least having considered and mentioned the causal assumptions required. After all, causal assumptions are the cornerstone on which causal inference relies, not simply an afterthought to be mentioned in the discussion after the analysis is done.

Accordingly, we suggest that causal software report the causal assumptions in the output. As an illustration, in CarefullyCausal, we print the causal assumptions immediately, by default, below the table of estimates so that the user is reminded that any causal interpretation given to the table of estimates requires those assumptions to be satisfied [15]. Rather than print a generic version of the assumptions, we apply the assumptions to the user’s context. We use simply stated versions of the assumptions tailored to the user’s analysis by replacing words such as “exposure” and “outcome” with the corresponding input variable names in the analysis. In addition to printing the assumptions below the estimates, in the saved output from the function, there are more detailed explanations provided, including a paragraph that contains all assumptions together (again, tailored to the variables in the analysis). This paragraph is a good starting point for a paragraph stating the assumptions in the methods section of the user’s report/manuscript. Shown below is an example of the output generated by CarefullyCausal:

To interpret these effects as causal, the following key assumptions must be satisfied:

[1] Conditional exchangeability requires that adjusting for "race, sex, education, smokeintensity, smokeyrs, wt71, exercise, active, age" is sufficientto eliminate all confounding and selection bias between "qsmk" and "wt82_71." See the covariate balance table ($Assumptions$exchangeability$covariate_balance) in the saved output and the corresponding explanations ($Assumptions$exchangeability$explanation).

[2] Positivity: is satisfied when both exposed and unexposed individuals are observed within every stratum of variables adjusted for (race, sex, education, smokeintensity, smokeyrs, wt71, exercise, active, age). This can be evaluated using the propensity plots saved in the output at $Assumptions$positivity$plots (or identically use the ps.plot() function), the table below ($Assumptions$positivity$ps_table) and the corresponding explanation at $Assumptions$positivity$explanation.

Note: PS=propensity score

   PS range for 1

   observed exposure: 0 0.0338, 0.6520

   observed exposure: 1 0.0685, 0.7709

[3] Consistency: implies that exposure ’qsmk’ must be sufficiently well-defined so that any variation within the definition of the exposure would notresult in a different outcome. See $Assumption$consistency for a more in-depth explanation and examples.

[4] No Interference: assumes that the exposure“qsmk”applied to one unit does not affect the outcome of other units.

[5] No measurement error: assumes that all variables were measured without substantial error, such that no substantial measurement bias is present. See $Assumptions$no_measurement_error for a further discussion.

[6] Well-specified models: assumes that any models used are well-specified meaning that they include all relevant non-linearities and/or statistical interactions.

To further support the assessment of the respective assumptions, CarefullyCausal provides indicative diagnostics for the causal assumptions when possible with accompanying explanations to help with interpretation. These include balance plots, balance tables, propensity score summary tables, and propensity score plots. Directly providing relevant diagnostics not only helps those less familiar with interpretation but also aims to enhance reporting of diagnostics in general, as it is reasonably common that studies do not report any diagnostics related to the assumptions or that it is reported incorrectly [1, 4, 5].

## Discussion

Statistical software has a role to play in the adoption of causal inference. Much as statistical software provides support to the user for correct statistical inference, it can also provide support for correct causal inference. We propose that support for applied causal inference should involve expanding the reported output to include: (i) the causal estimand, (ii) results from different estimators that rely on distinct modeling assumptions, and (iii) the causal assumptions alongside relevant diagnostics. We have demonstrated one possible implementation of this approach through our developed R function, CarefullyCausal. We want to emphasize, however, that this function is merely one possible implementation of our suggestions, with both the design and content open to discussion. For instance, while CarefullyCausal currently presents all output at once, a stepwise reporting approach might be more suitable. A two-step approach could first display the estimand, assumptions, and relevant diagnostics, encouraging the user to review these elements thoroughly before showing the statistical output. Alternatively, users could be required to pre-specify a primary estimator to mitigate *post-hoc* cherry-picking when multiple estimates are presented, though this may come at the cost of transparency in detecting bias arising from misspecification of the nuisance model. Such design considerations, along with determining what information would be most helpful to users, are worth investigating further.

On a higher level, the aim is to promote more comprehensive reporting of causal results in empirical research. The ideas discussed would ideally be integrated into both existing and new functions, packages, or software, each possibly adopting its own approach for implementing these ideas. However, we would be remiss to ignore the possibility that providing more causal information in software output could potentially disincentivize users from properly pursuing causal inference education because they can use the output as a crutch. For instance, we tailor the output and assumptions to the user’s context, but that also makes it easier to simply copy into a manuscript without properly understanding or considering the plausibility of the causal assumptions. This phenomenon also occurs with statistical inference [31, 32]. Such uncritical use would hopefully be caught by co-authors, at the peer review stage, or by an astute reader. It could be argued that, even in the worst-case scenario where a user would uncritically copy and paste the causal assumptions into their manuscript, this is still an improvement over a manuscript that does not include the causal assumptions at all. Even if the ultimate goal is to get researchers to discuss and scrutinize their assumptions, simply mentioning all assumptions is a step in the right direction.

There may also be a challenge of self-selection, as those users who are already aware that their question is causal would be more likely to use functions specifically designed for causal inference, including CarefullyCausal. The real challenge is reaching researchers who know their actual aim is causal but do not know how to address this appropriately, or even researchers who are trying to answer causal questions but are not aware of it because they have been trained to only use associational language. Unfortunately, there is no easy solution for this, except that the more statistical software includes information about causal inference, the more often researchers will be exposed to carefully thinking through the estimand, estimators, and assumptions in causal inference.

In summary, we hope to initiate a conversation about the role of software in causal inference. We have developed a function to demonstrate one possible form this could take: CarefullyCausal. Using such a function could enhance applied causal inference by not only making it more accessible and more careful but also by promoting transparency through increased reporting of assumptions. Including causal inference information in software output should by no means act as a substitute for existing resources for learning and using causal inference, but as a complement. The point is to question the current causal inference output conventions, regardless of the software used, and to show how providing more information on causal inference in statistical output may help make causal inference safer. We encourage current software designers and maintainers to reconsider the role of causal inference in their output, as it can lower the barriers to careful causal inference in applied research.

## Ethics approval

This research project does not require ethics approval as it uses non-identifying data that are freely available online.

## Supplementary Material

dyaf136_Supplementary_Data

## Data Availability

See data resource access, above.
