# Supplementary material for: Causal clarity in statistical software
Source: Int J Epidemiol. 2025 Jul 21;54(4):dyaf136. doi: 10.1093/ije/dyaf136 (PMC12281111; doi:10.1093/ije/dyaf136)
Supplement: dyaf136_Supplementary_Data [file dyaf136_supplementary_data.pdf]

Causal Clarity in Statistical Software: Supplementary Material

Maurice Korf[1,*], Nan van Geloven[2], Jesse H. Krijthe[3], and Jeremy Labrecque[1]

[1] *Department of Epidemiology, Erasmus MC University Medical Center, Rotterdam, The Netherlands*

[2] *Department of Biomedical Data Sciences, Leiden University Medical Center, Leiden, The Netherlands*

[3] *Department of Intelligent Systems, Delft University of Technology, Delft, The Netherlands*

[*] *Corresponding Author: Maurice Korf, m.korf@erasmusmc.nl*

Supplementary section A: results scoping review causal inference R packages

Table S1: Most downloaded causal inference R packages on the CRAN

Package	Method Class	Output	Source	Downloads[a]
`plm`[b]	regression	Only statistical output	[1]	3 525 698
`fixest`	regression	Only statistical output	[2]	467 785
`estimatr`	regression	Only statistical output	[3]	436 102
`riskRegression`	gcomp	Only statistical output	[4]	497 466
`modelbased`	gcomp	Only statistical output	[5]	305 418
`marginaleffects`	gcomp	Only Statistical output<br>Provides contrast in words	[6]	198 179
`ebal`	IPTW	Weights only	[7]	159 774
`CBPS`	IPTW	Propensity scores & weights<br>Balance diagnostics (e.g. plots)	[8]	154 600
`twang`	IPTW	Propensity scores & weights<br>Balance diagnostics (e.g. plots)	[9]	147 914
`grf`	DR	Only Statistical output<br>Statistical diagnostics	[10]	320 755
`tmle`	DR	Only Statistical output	[11]	69 403
`ltmle`	DR	Only Statistical output	[12]	53 674
`ivreg`[c]	IV	Statistical output<br>IV Diagnostic tests	[13]	219 134
`LARF`	IV	Only Statistical output	[14]	68 546
`ivmodel`	IV	Statistical output<br>IV Diagnostic tests & Plots	[15]	55 379
`mediation`	mediation	Statistical output<br>Result & sensitivity plot	[16]	599 932
`cit`	mediation	Only Statistical output	[17]	66 550
`medflex`	mediation	Statistical output<br>Result Diagnostics & Plots	[18]	60 095

[a] *See Supplementary section B for the complete protocol of the scoping review*
[b] *Note that* `plm` *is similar to* `lm` *and* `glm` *from* `stats` *but only* `plm` *is explicitly considered as belonging to the causal inference category on CRAN. Yet, they are all statistically oriented*
[c] *Note that* `ivreg,LARF,` *and* `ivmodel` *have respectively the fourth, fifth and sixth most downloads. However, the top three most downloaded packages for IV overlap with other method classes. Specifically, the top three most downloaded packages for IV are respectively* ***`fixest, estimatr,`*** *and* ***`grf`****. We include the fourth, fifth, and sixth most downloaded packages for IV to consider IV specific packages.*
[d] *gcomp=G-computation, IPTW=inverse probability of treatment weighting, DR=Doubly Robust, IV=instrumental variable*

Supplementary section B: protocol for selecting and reviewing R packages

The aim of this scoping review was to evaluate how many causal inference R packages reported the causal assumptions in their output. This provided us a broader perspective of what causal inference R packages do report and what they do not report in the output. We evaluated widely used causal inference related R packages that were on the Comprehensive R Archive Network (CRAN) at April 30th, 2024. The CRAN package repository contains 20644 packages [19]. The CRAN r-project website further provides a broad classification of R packages and classifies all the packages into 44 different categories where each category corresponds to specific tasks relevant for a certain topic [20]. Note that these 44 categories are referred to as *task views* on CRAN since the classification is done with respect to particular tasks that a package is designed for. One of those 44 categories is called `CausalInference` and provides an overview and a detailed subclassification of all R packages related to causal inference [21]. This category (task) includes 152 packages (Supplementary Figure S1). Within this Causal inference category (task), the CRAN subclassifies the R packages into 7 main causal inference themes, where we will focus on the theme called `Average treatment effect estimation and other univariate treatment effect estimates` [21]. This theme includes 103 R packages (not necessarily unique) and are further subclassified by the CRAN into 14 different methods. We will focus on 6 method classes, including: Regression Models, G-computation, Inverse Probability Treatment Weighting (IPTW), Doubly Robust Methods (DR), Instrumental Variable Methods (IV), and Mediation. This results in 65 causal inference related R packages to be eligible for evaluation, as shown in Table S2 for each of the six considered method classes. We will investigate the top 3 most downloaded packages for each method class, resulting in 18 R packages to be evaluated in terms of output produced. The decision to focus on these 6 method classes can be considered arbitrary, yet, we believe that these are the most widely used causal inference topics. This is demonstrated by the fact that these 6 method classes cover about 63% (65/103) of the causal inference packages related to the theme of `average treatment effect estimation`.

A schematic overview of the described protocol is presented in Figure S1. Moreover, the R code used to generate the content of Table S1 is provided in Supplementary Section C, allowing users to reproduce and update the descriptive information.

Table S2: All listed CRAN R packages under the category (task) `causal inference` and subcategory `average treatment effect estimation and other univariate effect estimation` [21]

**Methods**	**R Packages**
Regression Models	`fixest,estimatr,CausalGAM, sampleSelection, BCEE,borrowr,causaldrf,hdm,plm,alpaca`
G-computation	`gfoRmula,EffectLiteR,endoSwitch,riskRegression, marginaleffects,modelbased,stdReg`
IPTW	`WeightIt,MatchThem,PSweight,clusteredinterference, inferference,CBPS,twang,twangContinuous,sbw, optweight,ebal,mvGPS,maic,maicChecks`
Doubly Robust Methods	`AIPW,PSweight,doubleML,grf,causalweight,tmle drtmle,ctmle,ltmle`
Instrumental Variable Methods	`ivreg,ivmodel,bpbounds,grf,fixest,estimatr,DoubleML ivmte,LARF,icsw,ivdesc`
Mediation Analysis	`cfma,cit,MultisiteMediation,DirectEffects, medflex causalweight,twangMediation,mediation cfdecomp,paths,regmedint,gma,bmem`

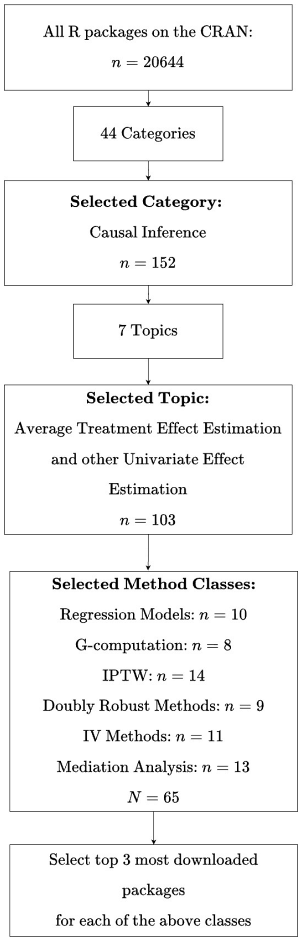

Figure S1: Schematic overview of protocol followed

Supplementary section C: R code to update and reproduce Table S1

The R script below allows to retrieve all R packages on the CRAN that are classified as `CausalInference` packages. In addition, the number of cumulative downloads are retrieved for each package using `cranlogs` where subsequently the top 3 downloaded packages per method class are shown in one list.

```
if (!require(c("ctv","cranlogs"))) {
  install.packages(c("ctv", "cranlogs"))
}
library(ctv) #ctv=Cran Task View
library(cranlogs)

# Get all package names in the category/task "CausalInference"
# Source: https://cran.r-project.org/web/views/
package_names <- as.vector(ctv::ctv("CausalInference")[["packagelist"]]$name)
length(package_names) #number of packages

# Retrieve package downloads using CRAN logs
# Beginning of CRAN logs is October 2012, hence to get total downloads we use
# that as start date ("from")
df <- cran_downloads(packages=package_names, from = "2012-10-01", to=Sys.Date())
downloads_df <- as.data.frame(aggregate(df$count, list(df$package), FUN=sum))
colnames(downloads_df) <- c("Package","Cumulative_downloads")

# Specify all CRAN packages to be considered, note that this needs to be specified
# manually from source: https://cran.r-project.org/web/views/CausalInference.html
packages_list <- list(
            regression_packages = c("fixest","estimatr","CausalGAM",
            "sampleSelection", "BCEE","borrowr","causaldrf","hdm",
            "fixest","plm","alpaca"),

            gcomp_packages = c("gfoRmula", "EffectLiteR", "endoSwitch",
            "riskRegression", "marginaleffects", "modelbased", "stdReg"),

            iptw_packages = c("WeightIt", "MatchThem", "PSweight",
```

```
            "clusteredinterference", "inferference", "CBPS", "twang",
            "twangContinuous", "sbw", "optweight", "ebal", "mvGPS", "maic",
            "maicChecks"),

            doublyrobust_packages = c("AIPW", "PSweight", "doubleML", "grf",
            "causalweight", "tmle", "drtmle", "ctmle", "ltmle"),

            iv_packages  = c("ivreg", "ivmodel", "bpbounds", "grf", "fixest",
            "estimatr", "DoubleML", "ivmte", "LARF", "icsw", "ivdesc"),

             Mediation_packages = c("cfma", "cit", "MultisiteMediation", "DirectEffects",
             "medflex", "causalweight", "twangMediation", "mediation",
             "cfdecomp", "paths", "regmedint", "gma", "bmem"))

# Check number of packages
length(unlist(packages_list)) #not necessarily unique
length(unique(unlist(packages_list))) #unique

# Select top X you would like to keep (we specify X=3)
# Output: list containing all top X most downloaded R packages for each in packages_list
top <- 3
top_list <- list()

for (i in 1:length(packages_list)) {
  ref_df <- downloads_df[downloads_df$Package %in% packages_list[[i]],]
  ref_df <- ref_df[order(-ref_df$Cumulative_downloads),]
  ref_df <- ref_df[1:top,]

  top_list[[names(packages_list)[i]]] <- ref_df}
```

Supplementary section D: CarefullyCausal R output

The `CarefullyCausal` R function can be downloaded from Github at: *https://github.com/mauricekorf/CarefullyCausal*. We present a comprehensive example on Github, where it is step-by-step shown how the function can be used and explain each of the arguments and its possibilities. We further provide in-depth technical details with respective references for those interested. Below we show screenshots of a minimal call and corresponding key output (again, in detail discussed on Github). The example is loosely based on the 'quit smoking' example from the *What if* book by Hernán and Robins, where the corresponding data are used here for illustration [22]. Note that the variables selected and therewith the numbers shown are for illustration purposes only.

```
output <- CarefullyCausal(wt82_71 ~ qsmk + race + sex + education + smokeintensity + smokeyrs + wt71 + exercise
                          + active + age,
                          data = df,
                          exposure = "qsmk",
                          family = "gaussian")
```

Figure S2: Minimal call required for `CarefullyCausal`

```
Estimand: Average Treatment Effect
E[wt82_71^qsmk=1]  -  E[wt82_71^qsmk=0]

Adjustment Set: race, sex, education, smokeintensity, smokeyrs, wt71, exercise, active, age
*Please see output at $Estimand_interpretation for details


Treatment effect:
                          Estimate Std. Error P-value S-value 95%.CI.lower 95%.CI.upper
qsmk1 outcome regression     3.381      0.441   0.000  44.858        2.517        4.246
qsmk1 IPTW                   3.318      0.494   0.000  35.198        2.351        4.286
qsmk1 S-standardization      3.381      0.432   0.000     Inf        2.511        4.204
qsmk1 T-standardization      3.448      0.483   0.000     Inf        2.475        4.367
qsmk1 TMLE                   3.370      0.494   0.000     Inf        2.401        4.339

Reference exposure level: 0


Please evaluate whether the difference beteen the lowest estimate: 3.3183 and highest: 3.4482 is of substance,
given the nature of the data. If so, evaluate the different modeling assumptions underlying each estimator.


To interpret these effects as causal, the following key assumptions must be satisfied:

[1] Conditional exchangeability requires that adjusting for "race, sex, education, smokeintensity, smokeyrs, wt71, exercise,
active, age" is sufficient to completely eliminate all confounding and selection bias between "qsmk" and "wt82_71". See the
covariate balance table ($Assumptions$exchangeability$covariate_balance) in the saved output and the corresponding explanati
ons ($Assumptions$exchangeability$explanation).

[2] Positivity: is satisfied when both exposed and unexposed individuals are observed within every stratum
of variables adjusted for ( race, sex, education, smokeintensity, smokeyrs, wt71, exercise, active, age ).
This can be evaluated using the propensity plots saved in the output at $Assumptions$positivity$plots
(or identically use the ps.plot() function), the table below ($Assumptions$positivity$ps_table)
 and the corresponding explanation found at $Assumptions$positivity$explanation. Note: PS=propensity score

                      PS range for 1
observed exposure: 0   0.0338, 0.6520
observed exposure: 1   0.0685, 0.7709

[3] Consistency: implies that exposure 'qsmk' must be sufficiently well-defined so that any variation within
the definition of the exposure would not result in a different outcome. See $Assumption$consistency
for a more in-depth explanation and examples.

[4] No interference: assumes that the exposure 'qsmk' applied to one unit does not affect the outcome of other units.

[5] No measurement error: assumes that all variables were measured without substantial error, such that
no substantial measurement bias is present. See $Assumptions$no_measurement_error for further explanation

[6] Well-specified models: assumes that any models used are well-specified, meaning that they include all
relevant non-linearities and/or statistical interactions
```

Figure S3: Printed output in R after running `CarefullyCausal`

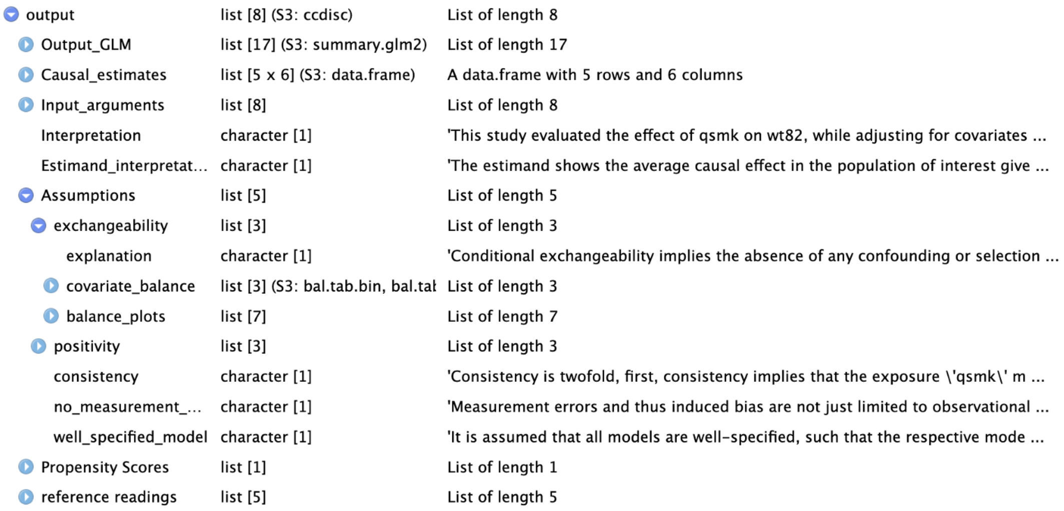

Figure S4: Saved output in R after running `CarefullyCausal`

References

1. Croissant Y and Millo G. Panel Data Econometrics in R: The plm Package. Journal of Statistical Software 2008; 27:1–43. DOI: 10.18637/jss.v027.i02
2. Bergé L. Efficient estimation of maximum likelihood models with multiple fixed-effects: the R package FENmlm. CREA Discussion Papers 2018
3. Blair G, Cooper J, Coppock A, Humphreys M, and Sonnet L. estimatr: Fast Estimators for Design-Based Inference. R package version 1.0.2, https://github.com/DeclareDesign/estimatr. 2024. Available from: https://declaredesign.org/r/estimatr/
4. Gerds TA, Ohlendorff JS, and Ozenne B. riskRegression: Risk Regression Models and Prediction Scores for Survival Analysis with Competing Risks. R package version 2023.12.21. 2023. Available from: https://CRAN.R-project.org/package=riskRegression
5. Makowski D, Ben-Shachar MS, Patil I, and Lüdecke D. Estimation of Model-Based Predictions, Contrasts and Means. CRAN 2020. Available from: https://github.com/easystats/modelbased
6. Arel-Bundock V. marginaleffects: Predictions, Comparisons, Slopes, Marginal Means, and Hypothesis Tests. R package version 0.19.0.2. 2024. Available from: https://marginaleffects.com/
7. Hainmueller J. ebal: Entropy Reweighting to Create Balanced Samples. R package version 0.1-8. 2022. Available from: https://CRAN.R-project.org/package=ebal
8. Fong C, Ratkovic M, and Imai K. CBPS: Covariate Balancing Propensity Score. R package version 0.23. 2022. Available from: https://CRAN.R-project.org/package=CBPS
9. Ridgeway G, McCaffrey DF, Morral AR, Cefalu M, Burgette LF, Pane JD, and Griffin BA. Toolkit for weighting and analysis of nonequivalent groups: a tutorial for the R TWANG package. Rand Santa Monica, Calif, 2022
10. Tibshirani J, Athey S, Sverdrup E, and Wager S. grf: Generalized Random Forests. R package version 2.3.2. 2024. Available from: https://CRAN.R-project.org/package=grf
11. Gruber S and Laan M van der. tmle: an R package for targeted maximum likelihood estimation. Journal of Statistical Software 2012; 51:1–35
12. Lendle SD, Schwab J, Petersen ML, and van der Laan MJ. ltmle: An R Package Implementing Targeted Minimum Loss-Based Estimation for Longitudinal Data. Journal of Statistical Software 2017; 81:1–21. DOI: 10.18637/jss.v081.i01

13. Fox J, Kleiber C, and Zeileis A. ivreg: Instrumental-Variables Regression by '2SLS', '2SM', or '2SMM', with Diagnostics. R package version 0.6-3. 2024. Available from: https://zeileis.github.io/ivreg/

14. An W and Wang X. LARF: Instrumental Variable Estimation of Causal Effects through Local Average Response Functions. Journal of Statistical Software 2016; 71:1–13

15. Kang H, Jiang Y, Zhao Q, and Small DS. Ivmodel: an R package for inference and sensitivity analysis of instrumental variables models with one endogenous variable. Observational Studies 2021; 7:1–24

16. Tingley D, Yamamoto T, Hirose K, Keele L, and Imai K. mediation: R Package for Causal Mediation Analysis. Journal of Statistical Software 2014; 59:1–38. Available from: http://www.jstatsoft.org/v59/i05/

17. Millstein J. cit: Causal Inference Test. R package version 2.3.1. CRAN, 2021. Available from: https://cran.r-project.org/package=cit

18. Steen J, Loeys T, Moerkerke B, and Vansteelandt S. medflex: An R Package for Flexible Mediation Analysis using Natural Effect Models. Journal of Statistical Software 2017; 76:1–46. DOI: 10.18637/jss.v076.i11

19. CRAN r-project. Contributed packages. Available at: https://cran.r-project.org/web/packages/. 30 April 2024, date last accessed.

20. CRAN r-project. CRAN Task Views. Available at: https://cran.r-project.org/web/views/. 30 April 2024, date last accessed.

21. Mayer I, Zhao P, Greifer N, Huntington-Klein N, and Josse J. CRAN Task View: Causal Inference. Version 2023-08-04. Available at: https://CRAN.R-project.org/view=CausalInference. 30 April 2024, date last accessed.

22. Hernán M and Robins J. Causal Inference: What If. Available at: https://miguelhernan.org/whatifbook. 20 April 2025, date last accessed
